# SPARK-MSNA: Efficient algorithm on Apache Spark for aligning multiple similar DNA/RNA sequences with supervised learning

**DOI:** 10.1038/s41598-019-42966-5

**Published:** 2019-04-29

**Authors:** V. Vineetha, C. L. Biji, Achuthsankar S. Nair

**Affiliations:** Department of Computational Biology and Bioinformatics, University of Kerala, Thiruvananthapuram, Kerala India

**Keywords:** Data mining, Software

## Abstract

Multiple sequence alignment (MSA) is an integral part of molecular biology. But handling massive number of large sequences is still a bottleneck for most of the state-of-the-art software tools. Knowledge driven algorithms utilizing features of input sequences, such as high similarity in case of DNA sequences, can help in improving the efficiency of DNA MSA to assist in phylogenetic tree construction, comparative genomics etc. This article showcases the benefit of utilizing similarity features while performing the alignment. The algorithm uses suffix tree for identifying common substrings and uses a modified Needleman-Wunsch algorithm for pairwise alignments. In order to improve the efficiency of pairwise alignments, a knowledge base is created and a supervised learning with nearest neighbor algorithm is used to guide the alignment. The algorithm provided linear complexity *O(m)* compared to *O*(*m*^2^). Comparing with state-of-the-art algorithms (e.g., HAlign II), SPARK-MSNA provided 50% improvement in memory utilization in processing human mitochondrial genome (mt. genomes, 100x, 1.1. GB) with a better alignment accuracy in terms of average SP score and comparable execution time. The algorithm is implemented on big data framework Apache Spark in order to improve the scalability. The source code & test data are available at: https://sourceforge.net/projects/spark-msna/.

## Introduction

Sequence alignment is used in bioinformatics to identify degree of similarity between biological sequences (DNA, RNA or protein), in understanding functional, structural and evolutionary relationship between them. Sequence alignment is of vital importance in molecular biology for studies involving molecular function prediction, evolutionary tree reconstruction and disease analysis. Needleman-Wunsch(NW) algorithm^1^, was one of the first implementations of dynamic programming in bioinformatics. It was an optimal sequence alignment algorithm with a tradeoff in computational time and space. For two sequences of length m and n, the time and space complexity is computed as *O*(*m* * *n*). By expanding the same algorithm for multiple sequence alignment (MSA), the complexity rises exponentially (*O*(*m*^*n*^) for *n* sequences of length *m*). Because of this high computational cost involved, NW algorithm cannot be used in multiple sequence alignment especially for large number of long sequences. Most popular implementations of MSA such as CLUSTAL^2^, MAFFT^3^, MUSCLE^4^ use approximation methods such as progressive and iterative approaches for faster execution and less memory utilization. Most of the algorithms implemented so far were derivatives of NW algorithm.

The improvements in DNA sequencing technology has led to an unprecedented increase in the amount of DNA and genome data being available for studies. Therefore, it is important to improve the scalability and performance of MSA tools. Cloud computing and the recently emerged big data technologies such as the new programming paradigm called MapReduce^5^, are effective ways to process huge volume of data of the order of petabytes and more. Implementation using big data frameworks for sequence alignment/mapping were reported in the literature for instance, Sadasivam *et al*., 2010^6^ presented a Hadoop based implementation of MSA using NW algorithm and Zhao *et al*., 2015^7^ presented Spark based implementation of local alignment.

Most of these research focused on improving the scalability of MSA using Big data frameworks, but not much research has happened in improving the MSA technique as such. These implementations are able to support large set of input sequences, but when it comes to massive DNA sequences, they are either unable to support or execute slowly when the count of sequences increase beyond 100. DNA sequences are highly similar compared to protein sequences. This similarity feature can be utilized to improve the alignment, and enable algorithm to tackle the volume and achieve better performance. Q. Zou *et al*.^8^ developed an algorithm which is proven to be highly efficient in performing MSA of similar DNA/RNA sequences. The algorithm uses centre star strategy along with trie tree data structure to improve the performance. Spark version of this algorithm HAlign II^9^, to support large volume of sequences reported promising results for similar DNA/RNA sequence alignment. MASC (Multiple Sequence Alignment Based on a Suffix Tree and Center-Star Strategy) is the implementation of same algorithm on CUDA architecture to obtain much faster performance for ultra large data sets^10^. PASTASPARK^11^ is another prominent implementation of MSA on Spark framework, which performs alignment based on SATé (Simultaneous Alignment and Tree estimation) and transitivity.

MSA could be further enhanced with a bounded dynamic programming algorithm^12^ at the pairwise alignment level. DDGARM, an improved NW algorithm^13^ for pairwise alignment has proved that, for highly similar sequences, optimal alignment can be achieved by filling only 10% of the matrix. Our algorithm uses the concept of Q. Zou *et al*., on progressive alignment, with modified NW algorithm for improved pairwise alignment. Key characteristics of the proposed algorithm include, (a) Suffix tree data structure for storing input sequences and identifying common substrings between sequences, (b) A knowledge base and nearest neighbor learning layer to guide the pairwise alignment, (c) Modified Needleman-Wunsch algorithm to perform pairwise alignments at each stage in order to reduce the memory and execution time of alignments and (d) Parallelization using MapReduce method for suffix tree construction and pairwise alignment to further improve the execution time.

## Methods

### Progressive Alignment

Progressive method is one of the basic alignment strategies used for MSA. It is known to provide reasonably good result and is the most widely used heuristic method for MSA^14^. Hence it is chosen as the core of our algorithm. The basic flow of progressive strategy is to prepare a guide tree and use series of pairwise alignments to align the sequences based on the branching order in the guide tree. The guide tree is formed based on the pairwise distance of sequences. Guide tree is formed in the order from shortest to longest distant pair. Initially the most closely related sequence pair is aligned and then the remaining sequences are aligned to the previous alignment until all sequences are aligned. Pairwise alignment is performed at each stage and it is refined at the final step while summing up the alignments. In the refinement step, the early gaps are revisited to adjust the penalties based on aligned sequences from other pairwise alignments. There are many MSA algorithms which uses modified forms of progressive methods^2^. Details about progressive alignment method along with pseudo code is given in Supplementary Material (Data S2).

The guide tree construction and pairwise alignment are the major contributors for the execution time and memory utilization in progressive alignment method. Use of data structure such as suffix tree which enables efficient storage and quick search of common substrings of the sequences help in improving the complexity of guide tree step. Similarly, the pairwise alignments are performed using the dynamic programming approach which becomes the most time consuming process when the sequences involved in the MSA are quite large. Bounded dynamic programming algorithm is used to enhance the performance of pairwise alignments.

From the suffix tree, common substrings can be rapidly extracted for highly similar DNA sequences. This leaves only the unmatched regions to be aligned. The modified pairwise alignment algorithm also provides substantial improvement in execution time and memory utilization as the similarity among the sequences increases.

### Suffix Trees to enhance alignment of similar sequences

Suffix trees greatly improve the performance of search on indexed string and hence are widely used in problems involving pattern matching, finding sub strings etc. Many existing alignment algorithms use suffix tree to identify matching substrings and there exists different algorithms for the construction of suffix tree^15–21^. Ukkonen suffix tree construction^15^ is followed in the implementation as it is superior in terms of time and space complexity^22^.

Each input sequence is partitioned in to equal size segments and these segments are used to construct the suffix tree. Suffix tree is characterized by a root and each edge is labeled by the nucleotide in the sequence. For any node *v*, the string formed by concatenating the edge labels from root to *v* is the path to that node, *path(v)*. Suffix tree is known to provide optimal search time^16,23^, which means, identifying the node *v*, which is closest to the root for a given pattern P, such that P is a prefix of *path(v)* can be performed in time linear to the length of P. All leaves in the subtree of the node *v* then represent the occurrences of the pattern P in string S.

If there are *n* DNA sequences with an average length of *m*, the time complexity for building a suffix tree for one sequence is *O*(*m*) (Lines 2–3 in Algorithm 1). After constructing the suffix tree, search the suffix tree for each segment of every sequence pair to identify the common substrings and matching segments. Searching the *n* sequences in the suffix tree costs *O*(*nm*) (Lines 5–7 in Algorithm 1). For the unmatched segments, record the percentage identity and difference in length if any. Since the sequences are partitioned to equal size segments, only the last segment of every sequence will be having different length. Only the unmatched segments are considered for pairwise alignments and the features ie; percentage identity and difference in length are used to extract learning from knowledge base. The guide tree for performing pairwise alignments are formed based on the similarity measure extracted for each sequence pair.

### Modified N-W algorithm for pairwise alignment

Our previous research had proved that, for pairwise alignments, optimal alignment can be achieved by populating only limited number of diagonals of the matrix^13^. The number of diagonals to be filled to obtain optimal alignment is not fixed in all cases. Hence, there is a need to find the minimum number of diagonals to be filled as a pre-requisite. This is done using dot plot approach. With some modifications to the dotlet^24^ algorithm, the number of diagonals to be filled can be obtained. Test results have proved that the similarity between sequences and the number of diagonals to be filled are inversely proportional. According to our previous research^13^, sequences with % identity (more than 50%) and difference in length (less than 25%) are reported to get a 50% improvement in memory utilization and execution time in pairwise alignments.

In our approach, pairwise alignment is performed only for the unmatched segments. As the similarity between sequences increases, the number of segments to be aligned reduces. The modified alignment algorithm further reduces the complexity as similarity increases. The most distant segment pair from the input sequences are chosen to identify the number of diagonals to be filled. This improves the execution time and at the same time ensures that all pairwise alignments provide optimal alignment as it would be the highest of diagonals count for the given input set. Even though this step involves only one pairwise alignment, this could become costly for very large sequences. Hence, a knowledge base is built with training data and a learning layer with nearest neighbor algorithm is used to extract knowledge out of knowledge base. With more learnings, the knowledge base becomes more accurate and would result in faster learning.

In the traditional dynamic programming based pairwise alignment, the complexity is *O*(*m*^2^)for sequence segments having length *m*. In our modified alignment approach, the complexity reduces to *O*(*m* * *k*) + *O*(2*m* * *d*), where k is the difference in length and d is the number of diagonals filled. In case of highly similar sequences, *k* → 0 and $d\ll m$, hence the complexity becomes *O*(*m*) compared to *O*(*m*^2^) in the case of traditional dynamic programming approach where we fill the entire matrix. The worst case complexity would be *O*(*m*^2^) as 2*m* * *d* becomes equal to *m*^2^, when the similarity between sequences decreases.

**Algorithm 1:**
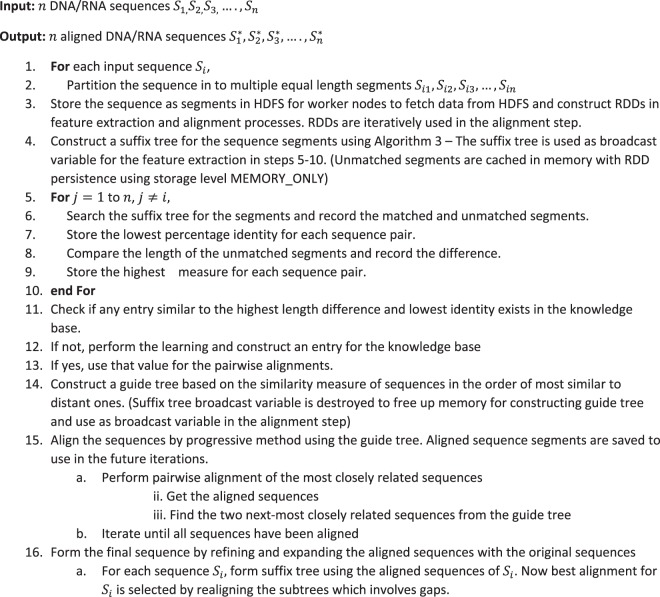
(Main flow):

### Supervised learning layer

Bounded dynamic programming for pairwise alignment is the key in our approach to achieve better performance. From the experiment results^13^, it is known that the number of diagonals to be filled depends on the similarity level and difference in length. Prior knowledge about the number of diagonals to be filled is a pre-requisite for the pairwise alignment step. Using training dataset, a knowledge base is built with the mapping of sequence similarity to number of diagonals. Sequence similarity measure (percentage identify and difference in length) for the most distant segments are used for extracting the knowledge from knowledge base. Nearest neighbor algorithm is used to identify the best matching entry from the knowledge base^25,26^. Less number of dimensions (percentage identity and length difference) for pattern recognition was the driving behind selecting nearest neighbor as the learning algorithm. More details about nearest neighbor algorithm is given in Supplementary Material (Data S3).

**Algorithm 2:**
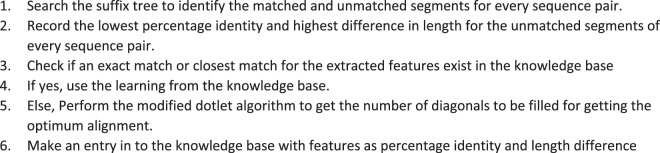
(Knowledge Base creation/learning).

**Algorithm 3:**
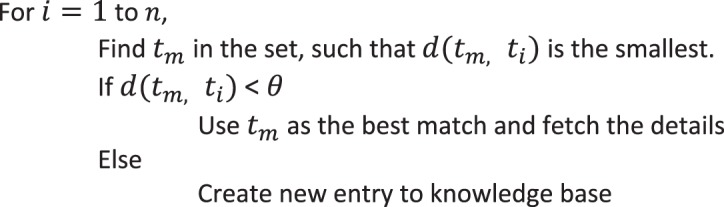
(Nearest Neighbor).

**Figure 1:**
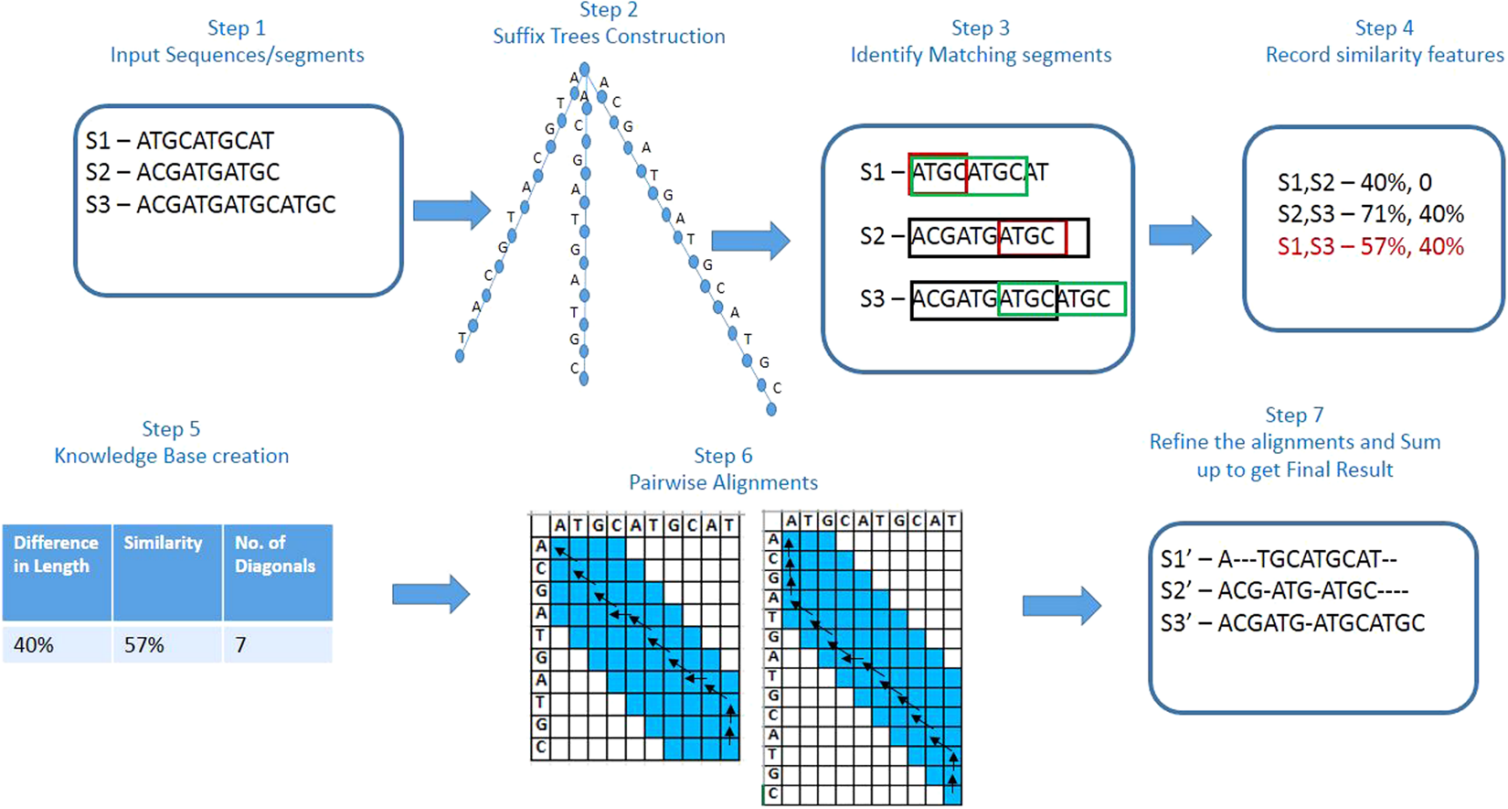
Sample flow of SPARK-MSNA algorithm.

The learning layer uses percentage identity and length difference as the features for classifying the input sequences. For each sequence pair, these features are extracted and the combination of highest value for difference in length and lowest value for identity are chosen for an input dataset. Then, it is matched with the knowledge base to identify the closest set. The algorithm initially checks for the exact match and in case of absence of exact match checks for the closest match (within a range of ±(2–3)%). Count of diagonals will be fetched for this closest match and that will be used for the pairwise alignment in the progressive MSA. Each time a new set of features are encountered, for which a closest match does not exist in the knowledge base, dotlet algorithm is executed to identify the number of diagonals. This learning is then entered in to the knowledge base for future alignments. More entries in the knowledge base would improve the performance and accuracy of the alignment. Figure 1 shows the flow of the algorithm with sample data.

### Parallel implementation with Spark

Parallel computation is implemented using MapReduce model at two stages in the algorithm. The suffix tree construction and the pairwise alignment of progressive method. MapReduce can be implemented using Hadoop or Spark. Due to the additional improvement in time provided by spark with its in-memory computation, spark is chosen as the MapReduce framework^27^. More details about MapReduce programming model is given in Supplementary Material (Data S4).

Although usage of suffix tree with Ukkonen’s algorithm results in linear time complexity, this could be costlier when sequences involved are quite large in size. Performance is further improved with parallel construction using MapReduce programming model^28^. The suffix tree is partitioned vertically and each partition is constructed independently. The prefixes generated from the vertical partitioning forms the key and its starting positions form the value. This key-value pair is processed using the map task and subtrees are constructed in parallel by compute nodes. Suffix tree construction from the subtree is combined with the map tasks in order to reduce the overhead of shuffle and reduce. Algorithm 4A shows the flow for the map function of suffix tree construction.

The pairwise alignment stage checks for matched segments and the unmatched segments alone are then taken for pairwise alignment. Pairwise alignment of segments is then executed in parallel using MapReduce model. Name of the sequence with segment index is the key and the sequence segment is the value for this map phase. Each compute node then performs the pairwise alignment using the modified pairwise algorithm. Result is then passed in the form of key-value pair where key is the sequence name with segment index and value is the aligned sequence. Aligned sequence segments for one pair of sequences are combined with the map task to avoid the overhead of reduce task. Algorithm 4B shows the flow of the map function for pairwise alignment.

**Algorithm 4A:**
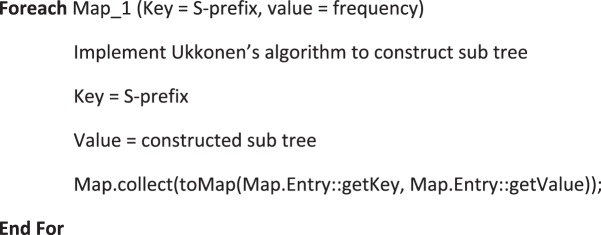
(Map function for Suffix tree construction).

**Algorithm 4B:**
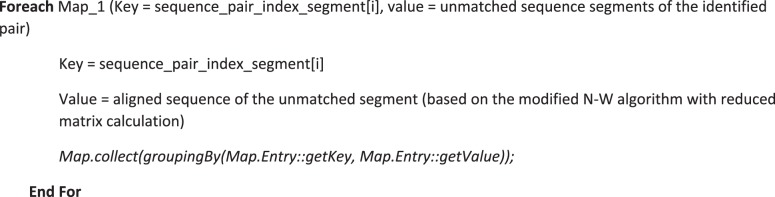
(Map function for pairwise alignment).

**Figure 2:**
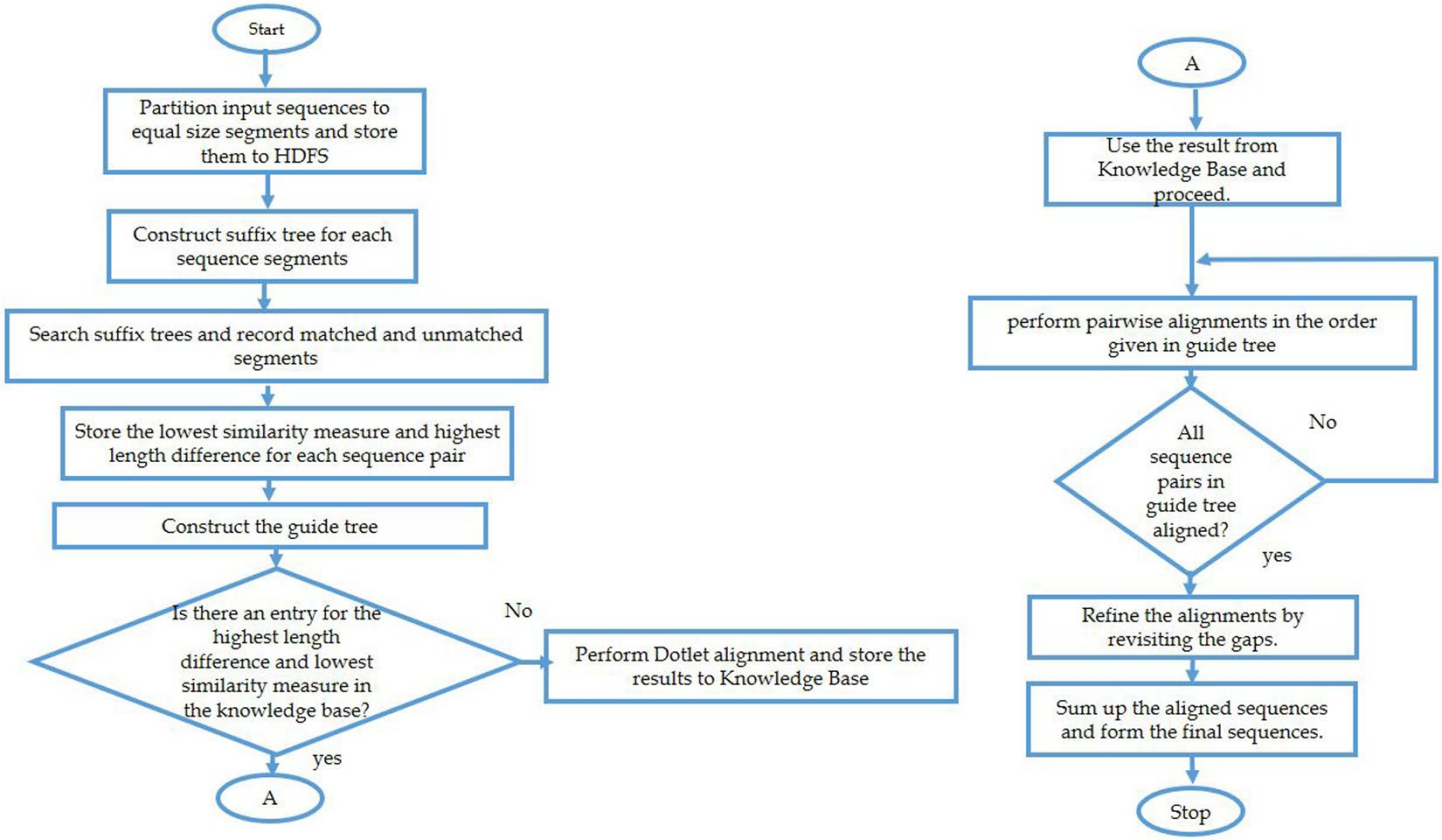
Flow chart of SPARK-MSNA algorithm.

Parallel execution does not improve the complexity of the algorithm but it helps in improving the execution time. When we have number of compute nodes equal to or more than the number of partitions to be processed, the execution time is equivalent to that of processing single partition plus an additional overhead for the reduce phase to construct the final result. In case the number of compute nodes are less, partition groups are formed and process the partition groups in parallel, for improved performance compared to sequential run. Spark framework reduces the network overhead by utilizing the data locality concept of MapReduce, but merging the scattered intermediate results to form the final result will always be there. But in case of large datasets, this additional overhead is much lower compared to the sequential execution or traditional distributed computing (OpenMP/MPI). Figure 2 shows the flow chart of the algorithm.

## Results and Discussion

### Test results on simulated data

Performance of the algorithm was tested using simulated data and couple of actual data sets. MSA algorithms supporting massive genome sequences are still in the evolving phase and hence there is a lack of benchmark dataset when it comes to large scale DNA MSA algorithms. The Balibase^29^ dataset which is considered as the golden benchmark for MSA is more suitable for protein sequences and does not provide benchmark for large DNA sequences. Details about real-world applications of MSA involving large genome sequences are given in Supplementary Material (Data S1). Simulated data with different levels of similarity was used to test the effectiveness of the algorithm. Test data was created by taking a portion of the human mitochondrial genome (NC_012920.1) as first sequence and then creating the second sequence with some random modifications in the first sequence. The similarity between sequences were first tested with traditional NW algorithm for correctness. Sequence datasets were prepared with 95%, 70%, 45%, 35% and 20% similarity and fixed size of 3.75 MB with maximum length 6580 bp and minimum length 6560 bp. Training data with 50 datasets of varying similarity range was prepared to build the knowledge base. Table 1 shows the snapshot of the knowledge base created for the test data.

**Table 1:** Sample knowledge base constructed for testing.

Difference in Length of sequences (%)	Similarity (%)	percentage of diagonals filled in the 2 × 2 matrix
0.30	99.20	0.15
0.20	99.30	0.14
0.40	98.00	0.16
0.30	98.10	0.15
0.10	95.90	0.18
0.23	97.30	0.17
0.34	98.20	0.16
0.35	96.40	0.18
0.70	99.00	0.4
1.20	99.00	0.3
5.80	75.00	6.2
25	50.00	20

**Figure 3:**
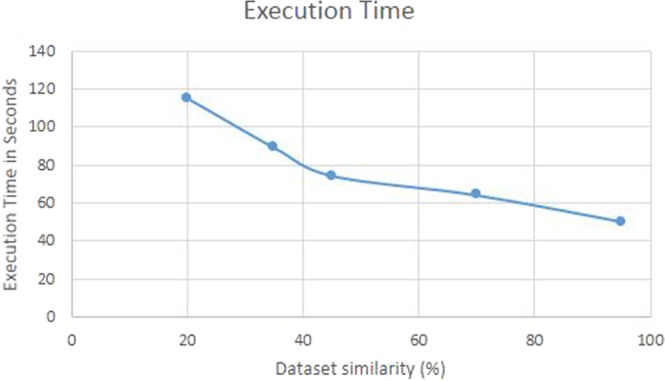
Execution time of SPARK-MSNA decreases as similarity of input sequences increase.

Prepared datasets were used to test the algorithm and identify the relationship of performance with similarity level. Testing was performed with and without knowledge base. Whenever the learning layer failed to match input data with existing entries in knowledge base, the algorithm performs dot plot step to gain the knowledge and make an entry in knowledge base. Table 2 shows the result of testing with simulated data. The results indicate that the algorithm delivers better performance as similarity between the input sequences increases. Figure 3 shows the decrease in execution time as similarity among input sequences increases. This is because, the number of segments to be aligned and the diagonals to be filled for alignment reduces as the similarity increases. With the knowledge base, the execution time and memory utilization reduces further as we do not have to perform the modified dotlet alignment to find out the number of diagonals to be filled. With large sequence data, the reduction in time due to removal of dotlet alignment would be more significant.

**Table 2:** Execution time taken by SPARK-MSNA for datasets with different similarity. Datasets were of equal size (3.75MB).

	Similarity (%)	Execution time (without knowledge base)	Execution time (with knowledge base)
Dataset 1	95	1 min 11 sec	50 sec
Dataset 2	70	1 min 31 sec	1 min 4 sec
Dataset 3	45	1 min 47 sec	1 min 14 sec
Dataset 4	35	2 min 5 sec	1 min 29 sec
Dataset 5	20	2 min 43 sec	1 min 55 sec

### Comparison with other tools

Most of the MSA algorithms compare the test results with other commonly used tools/algorithms. Test results of SPARK-MSNA are compared with HAlign, HAlign II and PASTASPARK. MAFFT & MUSCLE are used for comparing results of small data sets. Even though MASC has reported highly competitive performance in handling large volume of data, the underlying architectures are different for both implementations, as MASC is implemented on CUDA processor. The algorithms HAlign^8^ and HAlign II^9^ have reported considerable improvement in performance specifically in addressing large scale DNA sequence data and our work has been inspired from HAlign; so, we have used same datasets used by HAlign to test the performance of our algorithm – human mitochondrial genomes (mt genomes) and 16 s rRNA. Dataset from PASTASPARK 200k RNASim is also included in the test data.

**Figure 4:**
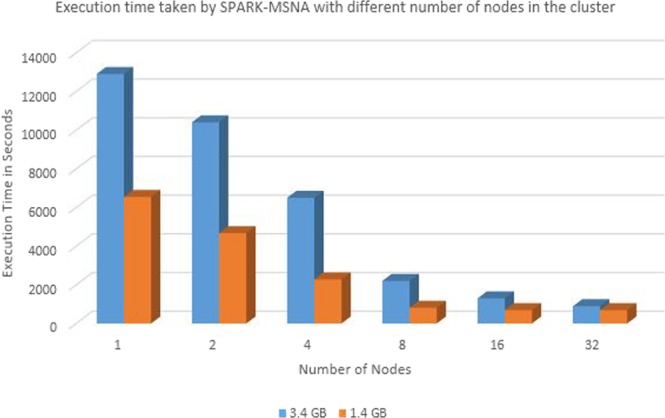
Improvement in execution time of SPARK-MSNA with more number of nodes.

**Figure 5:**
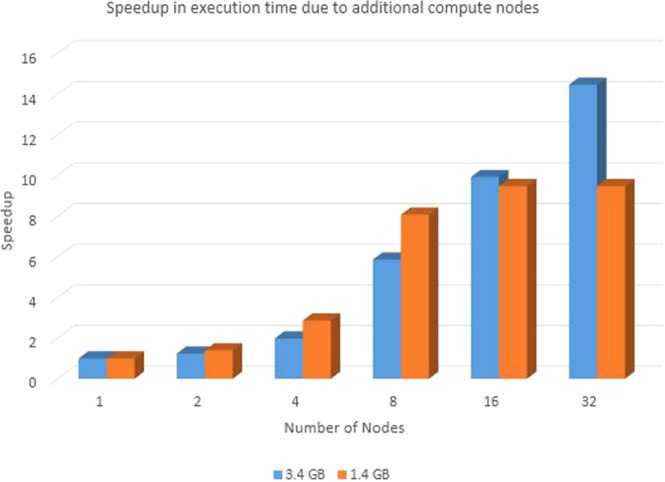
Speedup in execution time due to additional compute nodes.

**Figure 6:**
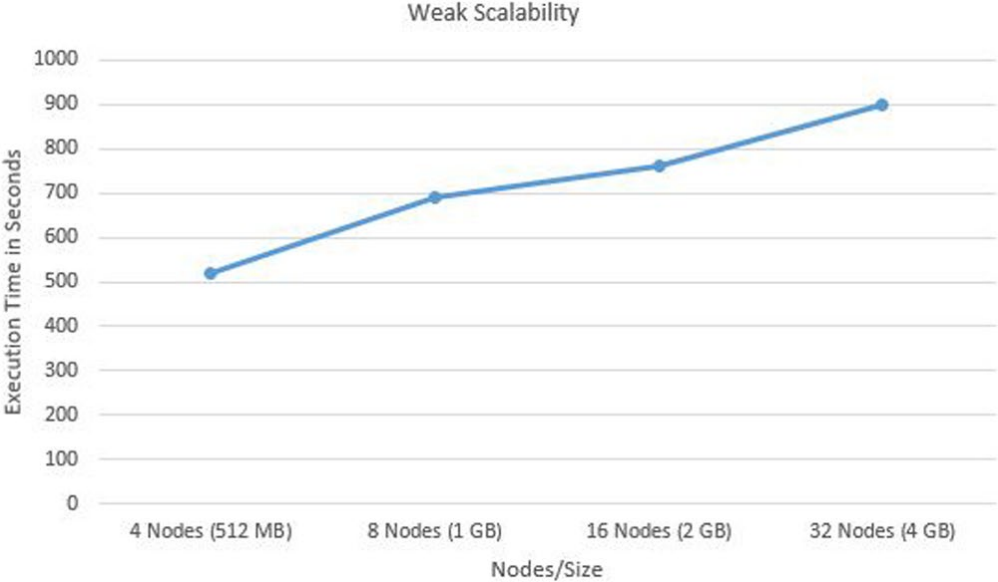
Weak scalability of SPARK-MSNA.

**Figure 7:**
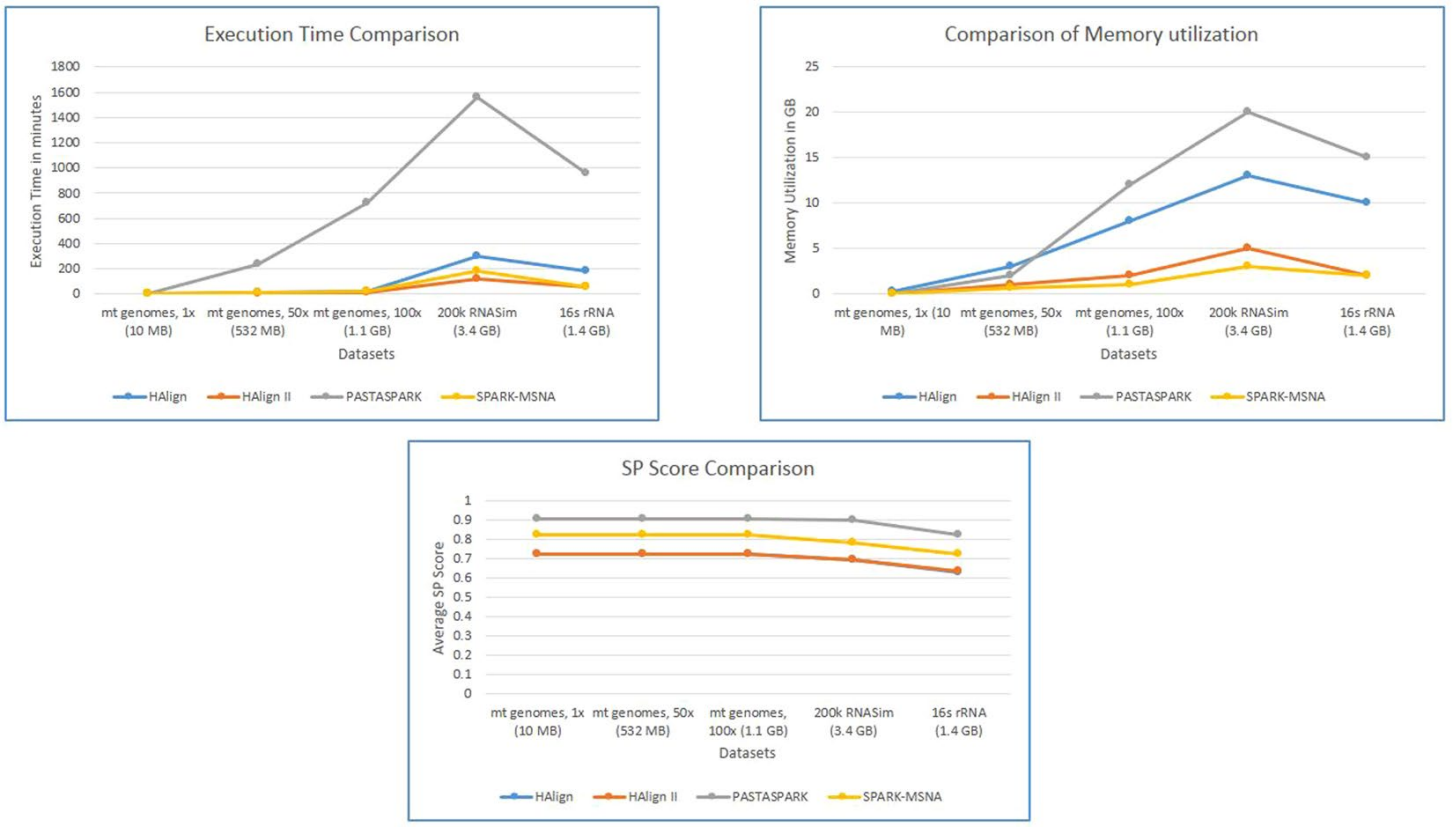
Performance comparison of SPARK-MSNA with other algorithms.

The human mitochondrial genome dataset is a sample for highly similar dataset. The dataset contains 672 human mitochondrial genomes with maximum length 16579 bp and minimum length 16556 bp. The percentage identity is >97% for this dataset. 200k RNASim dataset is used as dataset with moderate level of similarity with minimum sequence length as 748 and maximum sequence length as 1836. 16 s rRNA dataset is used for testing the performance on less similar sequence set. It has minimum length 807 bp and maximum length 1629 bp. Details of test datasets are provided in Table S1 of Supplementary Material. In order to compare results with other tools, tests are performed on single node cluster and multi node cluster. Spark cluster was set up on single node with 3.6 GHz 4 core CPU, 64 bit Ubuntu OS and 64 GB memory. In order to test the improvement due to parallel implementation, SPARK-MSNA was tested with more number of nodes. Figure 4 shows the execution time taken by SPARK-MSNA with 1, 2, 4, 8, 16 and 32 nodes. Large data sets of 1.4 GB and 3.4 GB are used for testing the improvement in execution time with number of nodes. Multi node cluster set up is used to compare performance with HAlign II. A cluster of 12 servers with intel Xeon E5-2620 processor with 8 cores and Spark 2.3.0 were used for the testing. Figure 5 shows the speedup of execution time due to additional nodes and Fig. 6 shows the weak scalability of the algorithm. Table S2 of Supplementary Material shows the test results of various algorithms using test datasets. Figure 7 shows the performance comparison of SPARK-MSNA with HAlign, HAlign II and PASTASPARK. MAFFT & MUSCLE had limitations in processing datasets of size more than 1 GB. Considering the high volume of genome sequence data generated by NGS techniques and the predicted transition to personalized and precision medicine, there is a pressing need on MSA tools/algorithms to support data sets of hundreds of GBs/TBs. SPARK-MSNA provided better optimum results with better memory utilization & average SP score compared to HAlign II with slightly high execution time.

Average SP score is used for comparing the alignment accuracy. SP score is calculated as the number of pairs of residues correctly aligned. The score is calculated as

$${\Sigma }_{i=1}^{{M}_{r}}{S}_{i}\,/\,{\Sigma }_{i=1}^{{M}_{r}}{S}_{ri}$$

where, M is the length of the sequence, *M*_*r*_ is the length of the reference sequence, *S*_i_ is the score of the *i*th column and *S*_*ri*_ is the score of *i*th column in reference sequence. *S*_i_ is calculated as normalized total score of column *i*, with pair value calculated as 2 if residues are aligned, 1 if one of the alignments has a gap and 0 otherwise. The test results show that SPARK-MSNA performs better in terms of memory utilization and accuracy, but has increased execution time compared to HAlign II. Progressive alignment and the refinement step increases the execution time, but that helps in achieving a better alignment. The reduced matrix alignment guided by knowledge base leads to reduction in memory utilization. MAFFT and MUSCLE provide better average SP score compared to SPARK-MSNA, but they are unable to handle large volume of dataset. MAFFT and MUSCLE failed to deal with datasets of size more than 1 GB. PASTASPARK is able to handle the large volume of data, but the execution time is much higher compared to HAlign II and SPARK-MSNA.

The modified Needleman-Wunsch algorithm for pairwise alignment of unmatched segments plays a crucial role in reducing the memory utilization for SPARK-MSNA. In the pairwise alignment step, HAlign II uses complete 2 × 2 matrix for alignment, whereas, SPARK-MSNA uses limited diagonals (approx. 2% of diagonals) to calculate the alignment. This modification provides up to 50% reduction in the memory utilized (depending on sequence similarity)^13^. The trade-off is between execution time and alignment accuracy. SPARK-MSNA provides a better balance between the two by providing an optimum performance in terms of computational time and memory while retaining an average SP Score close to MAFFT.

The knowledge base guides the bounded dynamic programming for pairwise alignment. Hence, a rich knowledge base results in better performance and better accuracy. For highly similar sequences, the improvement is highly significant as very less number of matrix cells (diagonals) are included in the alignment. As the similarity decreases, the number of matrix cells needed in the alignment increases and for highly different sequences, complete matrix is needed in the alignment, which makes it similar to normal dynamic programming. This is evident in the test result of 16 s rRNA dataset, where the memory utilization is similar to that of HAlign II.

In order to test the efficiency of knowledge base, we added the knowledge base layer (training layer) to HAlign II and tested the same using mt. genome and 16 s rRNA datasets. Table S3 of Supplementary Material shows the test results. Results show that, knowledge driven bounded dynamic programming helps in achieving improved execution time and memory utilization. Average SP score remains same as HAlign II as the underlying alignment strategy remains the same in both algorithms (Centre star). This shows the importance of knowledge driven algorithms, which can learn from their experiences are key to improving the performance of MSA.

### Complexity Analysis

The most important feature of SPARK-MSNA is the improved time and space complexity. The first stage of SPARK-MSNA is construction of Suffix tree. The Ukkonen’s algorithm using MapReduce model is adapted here. For one DNA sequence of length m, the time complexity for building suffix tree is *O*(*m*). For *n* sequences, the complexity is *O*(*nm*). The second stage is searching the suffix tree for all possible pair combinations of *n* DNA sequences. The search would incur a cost of *O*(*m*) for one sequence pair and we have *nC*2 pairs. So, the complexity of search becomes *O*(*n*^2^*m*). Building guide tree based on the similarity measure obtained from search is the next step. This incurs a linear cost of *O*(*n*). Due to less number of dimensions and cardinality involved in pattern matching, the complexity of learning layer becomes *O*(*k*).

Pairwise alignment of unmatched segments of sequence pairs is the next step. Since we are adopting the modified Needleman-Wunsch algorithm, the complexity becomes $O(kx)+O(2kd)$, where *k* is the segment length, *x* is the difference length of segments involved in pairwise alignment and *d* is the number of diagonals to be populated. For highly similar sequences, $x\to 0\,and\,d\ll k$, hence the complexity will be *O*(*k*). In traditional dynamic programming approach, the complexity is *O*(*k*^2^). The last step of summing up the alignment results to form the final alignment would incur a cost of *O*(*nm*). The learning step involves one pairwise alignment of the most distant segment pair and it incurs a cost of *O*(*k*^2^), if the learning is not available in the knowledge base.

Building the knowledge base is not part of the main flow of the algorithm. It is part of the training phase and hence it does not add to the overall complexity of the algorithm. Whenever the appropriate learning is missing in the knowledge base, the learning step is implemented to enhance the knowledge base. Modified dotlet algorithm is performed to get the number of diagonals. The alignment is performed on the most distant segment of sequences and in such scenarios, there would be an additional *O*(*m*^2^) added to the complexity of the algorithm, where *m* is the sequence length.

The overall complexity of SPARK-MSNA is $O(m)+O({n}^{2}m)+O(n)+O(k)+O(k)+O(nm)$. Considering $n\ll m$, the best case complexity is *O*(*m*). As the similarity between sequences decreases, number of unmatched segments and the number of diagonals to be populated for alignment increases, this will make the worst case complexity as *O*(*m*^2^). Same is the case when learning step is involved.

## Discussion

In this work, we have focused on improving the efficiency of MSA involving large DNA sequences by utilizing its similarity feature and improving the performance with learning layer and parallel execution. The test results and complexity shows that, SPARK-MSNA provides a better trade-off compared to other MSA tools/algorithms in handling similar large scale DNA/RNA sequences. SPARK-MSNA provides better alignment and memory utilization with a comparable execution time with large sequences. In best case scenario, SPARK-MSNA reduces the memory utilization up to 50% along with better alignment compared to HAlign II. In worst case scenario, where we cannot reduce the number of matrix cells to be processed in the pairwise alignments, the complexity remains similar to HAlign II. Test results with learning layer added to centre star approach shows that a knowledge driven approach helps in improving the performance in terms of execution time and memory. Knowledge driven algorithms, which can learn from experience and use the learnings in future alignments are instrumental in handling large scale datasets.

The proposed knowledge base uses only similarity feature for learning. Adding more features in knowledge base and alignment approach to utilize those additional features could provide a better result in future. RDD persistence using kyro serialization instead of raw data format for improved memory utilization is also planned as a future enhancement.

## Supplementary information

Supplementary Material
