# Supplementary material for: SPARK-MSNA: Efficient algorithm on Apache Spark for aligning multiple similar DNA/RNA sequences with supervised learning
Source: Sci Rep. 2019 Apr 29;9:6631. doi: 10.1038/s41598-019-42966-5 (PMC6488671; doi:10.1038/s41598-019-42966-5)
Supplement: Supplementary file 1 — Supplementary Material [file 41598_2019_42966_MOESM1_ESM.pdf]

# SPARK-MSNA: Efficient algorithm on Apache Spark for aligning multiple similar DNA/RNA sequences with supervised learning

Vineetha V., Biji C.L. and Achuthsankar S. Nair

## Supplementary Material

**Data S1 – Real world applications of MSA involving large genome sequences:**

- Multiple Sequence Alignment (MSA) forms the basis for
  - Detecting similarities between sequences (identify closely or distantly related)
  - Detecting conserved regions or motifs in sequences
  - Detecting key functional residues
  - Detecting structural homologies
  - Predicting secondary or tertiary structures
  - Inferring the evolutionary history of a protein family
- MSA is also used in:
  - Whole-genome sequencing (WGS), whole-exome sequencing (WES), and/or targeted sequencing, which are applied to biomedical study and medical practice to identify disease- and/or drug-associated genetic variants to advance precision medicine.
  - Phylogenetic reconstruction - Evolutionary analysis of bacterial and viral genomes (eg; analyzing influenza virus DNA)
  - Genome-wide association study (GWAS) approaches used to study genetic disorders and clinically relevant complex traits
  - Genome analysis toolkit (GATK) pipeline, used to elucidate new biological pathways from analysis of protein-protein interaction networks.
  - Pharmaceutical industry to identify molecular functions and pathways associated with a disease and to improve the drug discovery pipeline.
- The iPTOL project (iPlant Collaborative, 2013) and the Thousand Transcriptome project (1KP) (Wong, 2013) which analyze more than 100,000 sequences.

**Data S2 – Progressive Alignment Method:**

Progressive alignment is one of the commonly used heuristics in MSA. The basic algorithm for progressive alignment is as follows:

**Input:** $n$ DNA/RNA sequences $S_{1,}S_{2,}S_{3,} \dots .,S_n$

**Output:** $n$ aligned DNA/RNA sequences $S_1^*,S_2^*,S_3^*,\dots .,S_n^*$

1. **For** each pair of input sequence $S_i,S_j$,
2. Compute pairwise distance scores of $(S_i,S_j)$
3. Construct a guide tree which has sequences with lowest distance score at the root and similar sequences are nearer in the tree.
4. Align the sequences one by one according to the guide tree

Different methods can be used for calculating the distance scores of sequences and aligning the sequences.

**CLUSTAL-W** is a popular progressive alignment method used for MSA. It uses gap positions and match positions in sequences to calculate the distance and constructs a guide tree.

**T-COFFEE** is another progressive algorithm which uses output from CLUSTAL and a local alignment program LALIGN to find multiple regions of local alignment between two sequences. The resulting tree is used for progressively aligning the sequences.

**MAFFT** is another algorithm where Fast Fourier Transform is used to cluster the sequences and then the sequences are progressively aligned based on the clusters.

**Data S3 – Nearest Neighbor algorithm:**

Nearest neighbor algorithm is a simple and easy to implement supervised learning algorithm used in classification and regression problems.

Nearest neighbor algorithm works on the assumption that similar things exist in close proximity. It calculates the distance between 2 points and based on the distance it identifies the similar data points. This is a lazy algorithm, meaning no explicit training or only minimal training is needed. The entire data points or training data is needed for test phase. The steps or pseudo code for the algorithm is as follows:

Step 1: Prepare the training data or knowledge Base

Step 2: Classify the query data $X$ by finding the training example $(X_i,Y_i)$ that is nearest to $X$ according to Euclidean distance:

$$||x - x_i|| = \sqrt{\sum_j (x_i - x_{ij})^2}$$

In the proposed algorithm, nearest neighbor algorithm is used to identify the data point from knowledge base which is closest to the query data and the fetched data point is used to determine the number of diagonals to be processed for pairwise alignments.

**Data S4 – MapReduce programming model:**

Google's MapReduce programming model is designed for processing large datasets in highly parallel manner. MapReduce model works on the following basic concepts:

- Data parallelism
- Computation of key/value pairs from pieces of inputs
- Iteration over input
- Grouping of intermediate results by key
- Iteration over resulting groups
- Reduction of groups to form final result

MapReduce model provides powerful parallelism and highly simple in terms of abstracting the issues of distributed and parallel programming from programmer. The model takes care of load balancing, network performance and fault tolerance.

The input data is split in to chunks and it is stored in multiple data nodes. Processing needs to be implemented in MapReduce model such that map phase takes care of iterative processing of pieces of input data in key-value pairs. Reduce phase takes care of grouping the final result based on key-value pair.

The below figure shows the MapReduce execution overview. Any job is divided in to multiple tasks and each task is carried out by different nodes in the cluster. In order to improve the processing speed, each node processes data stored locally on that node. Each job is divided in to two phases, Map & Reduce. Mapper consists of parallel functions each processing a portion of input data. MapReduce engine attempts to ensure that Mappers run on nodes which hold the portion of data locally. Mapper reads data in the form of key/value pair and outputs zero or more key/value pairs. After Map phase is over, the intermediate results from nodes are combined together to form the final result. This phase is known as Reducer. The data nodes perform the map phase and name node executes the reducer phase. The concept is taking processing to the data rather than taking data to processor. Process flow management is taken care by MapReduce engine, so that user can focus only on writing the Map and Reduce functions.

A key concept in MapReduce model to address the network performance challenge in distributed computing is the exploitation of locality of data. Quite different from traditional distributed computing where data is brought to the processing job, here MapReduce code is sent to the data nodes on which data related to the MapReduce code is available. Thus the usage of network is limited, as much as possible, to merge scattered results.

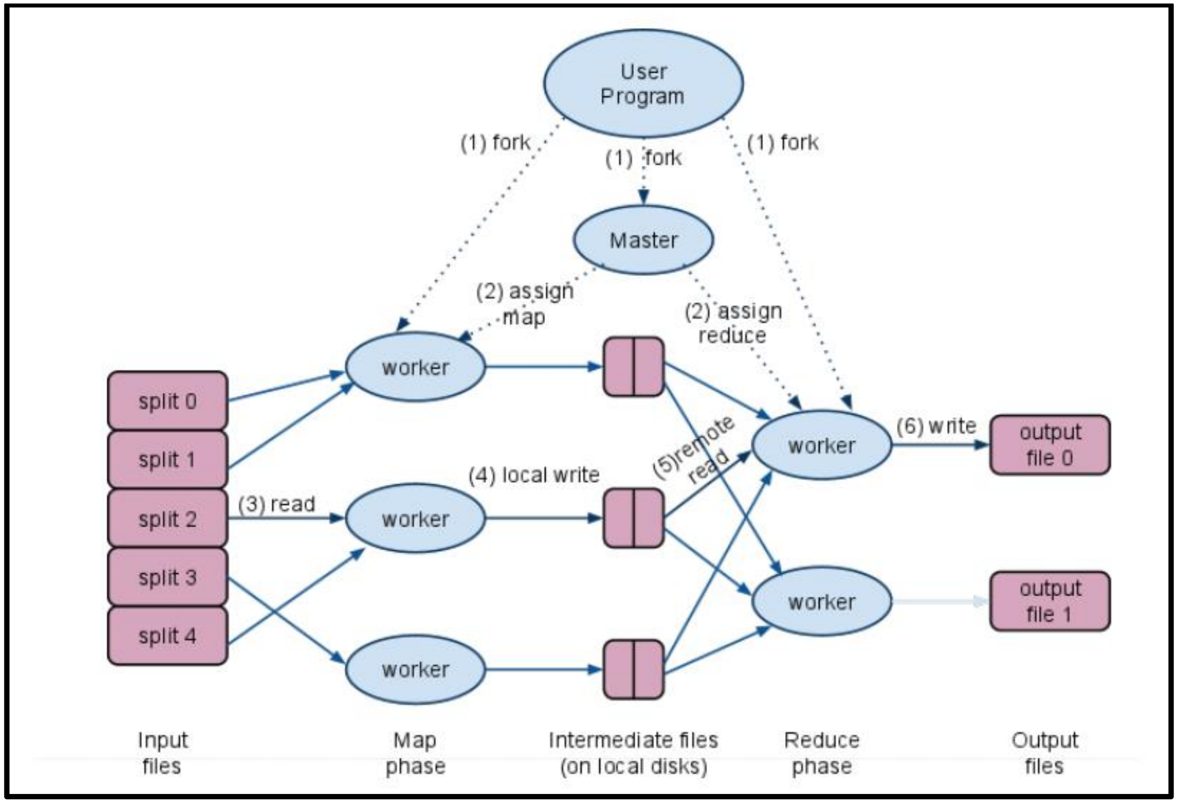


*(source: https://storage.googleapis.com/pub-tools-public-publication-data/pdf/36249.pdf)*

## Test Data and Results

- **Test datasets were selected based on significance and to include different levels of similarity.**

To evaluate the computational complexity, a detailed analysis was performed using sample datasets consisting of human mitochondrial genomes (mt. genomes), 16s rRNA (Zou, Q et al., 2015) and 200k RNASim (Jose M. Abuin et al., 2017). Human mitochondrial genomes and 16s rRNA datasets were chosen based on their significance in MSA. They also represent sequences with high and low similarity levels. With an aim to test the performance of our algorithm on varied similarity levels, 200k RNASim dataset was chosen to include sequences with moderate level of similarity. Human mitochondrial genome (mt. genomes) dataset was of size 10MB and to test the performance on large scale data, datasets were prepared by duplicating the sequences 50 and 100 times.

**Table S1. Input datasets used for testing**

| Dataset | Minimum Length (bps) | Maximum length (bps) | Average length | Similarity | Size |
|---|---|---|---|---|---|
| Human mitochondrial genome (1x) | 16556 | 16579 | 16,569.7 | ~97% | 10 MB |
| Human mitochondrial genome (50x) | 16556 | 16579 | 16,569.7 | ~97% | 532 MB |
| Human mitochondrial genome (100x) | 16556 | 16579 | 16,569.7 | ~97% | 1.1 GB |
| 200k RNA sim | 748 | 1836 | 1556 | ~60% | 3.4 GB |
| 16s rRNA | 807 | 1629 | 1388.5 | ~20% | 1.4 GB |

**Table S2. Performance comparison of SPARK-MSNA with other algorithms**

| | mt genomes, 1x (10 MB) | | | mt genomes, 50x (532 MB) | | | mt genomes, 100x (1.1 GB) | | | 200k RNASim (3.4 GB) | | | 16s rRNA (1.4 GB) | | |
|---|---|---|---|---|---|---|---|---|---|---|---|---|---|---|---|
| | Execution Time | Memory | SP Score | Execution Time | Memory | SP Score | Execution Time | Memory | SP Score | Execution Time | Memory | SP Score | Execution Time | Memory | SP Score |
| MAFFT | 1 m 41 sec | ~ 100 MB | 0.926 | 10 hr 15 m | ~ 2 GB | 0.926 | – | – | – | – | – | – | – | – | – |
| MUSCLE | 45 m 23 sec | ~ 8 GB | 0.951 | – | – | – | – | – | – | – | – | – | – | – | – |
| HAlign | 2 m 15 sec | ~ 300 MB | 0.722 | 14 m 15 sec | ~ 3 GB | 0.722 | 26 m 35 sec | ~ 8 GB | 0.722 | 5 hr 52 m | ~13 GB | 0.692 | 3 hr 20 m | ~ 10 GB | 0.631 |
| HAlign II | 14 sec | ~ 100 MB | 0.723 | 5 m 22 sec | ~ 1 GB | 0.723 | 10 m 25 sec | ~ 2 GB | 0.723 | 2 hr 48 m | ~ 5 GB | 0.692 | 59 m 42 sec | ~ 2 GB | 0.633 |
| PASTASPARK | 2 m 8 sec | ~100 MB | 0.908 | 4 hr 20 m | ~2 GB | 0.908 | 12 hr 30 m | ~12 GB | 0.908 | 26 hr 38 m | ~20 GB | 0.898 | 16 hr 45 m | ~15 GB | 0.826 |
| SPARK-MSNA | 1 m 20 sec | ~ 80 MB | 0.821 | 9 m 36 sec | ~ 700 MB | 0.821 | 19 m 18 sec | ~ 1 GB | 0.821 | 3 hr 35 m | ~3 GB | 0.783 | 1 hr 49 m | ~ 2 GB | 0.725 |

Results showed that, execution time and memory utilization increases based on the size of input dataset and the similarity feature. SPARK-MSNA provided best values for memory utilization. Execution time is slightly high when compared to HAlign II, but that is compensated with a better average SP score value. Thus, SPARK-MSNA provided a better trade-off between accuracy & computational cost as it provided better average SP score and less memory utilization compared all other algorithms. Less memory utilization is due to the reduced matrix processing during pairwise alignments. Execution time is slightly higher compared to HAlign II due to progressive alignment involved.

SPARK-MSNA provided better memory utilization and alignment accuracy in terms of average SP score compared to HAlign II, but execution time is slightly higher. This is due to the progressive method that we implement for alignment which involves multiple iterations.

In order to test the efficiency of having a learning layer to guide the algorithm (knowledge base), we added the knowledge base layer (training layer) to HAlign II and tested the same using mt. genome and 16s rRNA datasets. Table S3 shows the test results.

Results show that, knowledge base driven bounded dynamic programming helps in achieving improved execution time and memory utilization. Average SP score remains same as HAlign II as the underlying alignment strategy remains the same in both algorithms (Centre star). This shows the importance of knowledge driven algorithms, which can learn from their experiences are key to improving the performance of MSA

**Table S3. Performance comparison of SPARK-MSNA using Centre Star**

| | 1.1 GB (mt genomes, 100x) | | | 1.4 GB (16s rRNA) | | |
|---|---|---|---|---|---|---|
| | Execution Time | Memory | SP Score | Execution Time | Memory | SP Score |
| Halign | 26 m 35 sec | ~ 8 GB | 0.722 | 3 hr 20 m | ~ 10 GB | 0.631 |
| Halign II | 10 m 25 sec | ~ 2 GB | 0.723 | 59 m 42 sec | ~ 2 GB | 0.633 |
| SPARK-MSNA (Centre Star) | 9 m 32 sec | ~ 1 GB | 0.723 | 59 m 40 sec | ~ 2 GB | 0.633 |
